# A cross-sectional survey of attitudes towards education in implant dentistry in the undergraduate dental curriculum

**DOI:** 10.1186/s40729-020-00224-8

**Published:** 2020-07-08

**Authors:** Ramona Schweyen, Bilal Al-Nawas, Christin Arnold, Jeremias Hey

**Affiliations:** 1 Universitätspoliklinik für Zahnärztliche Prothetik, Martin Luther Universität Halle-Wittenberg, Magdeburger Str. 16, 06112 Halle, Saale Germany; 2 Universitätsklinik und Poliklinik für Mund-, Kiefer- und Gesichtschirurgie, Plastische Operationen, Johannes-Gutenberg Universität Mainz, Augustusplatz 2, 55131 Mainz, Germany

**Keywords:** Dental curriculum, Implant dentistry, Learning objective, Undergraduate education

## Abstract

**Background:**

An ongoing debate in dental education is whether implant dentistry, as a multidisciplinary domain, should be integrated into the undergraduate curriculum. The aim of the present study was to evaluate the perspectives of novices, clinical educators, and experienced dentists with regard to the importance of theoretical and practical implant dentistry teaching content in undergraduate dental education. The specific objective was to determine whether a consensus could be found concerning aspects of theoretical knowledge, implant position planning, implantation, prosthetic treatment procedures, postoperative care, and prerequisite experiences that should be provided in undergraduate dental education.

**Results:**

A positive consensus existed in terms of theoretical education, assistance in surgical and prosthodontic procedures, implant planning and restoration in straightforward cases (i.e., posterior single crowns and bridges, overdentures on nonconnected implants), and postoperative care. A negative consensus existed for bone augmentation. Implantation was supported by novices (i.e., students and graduates). In addition, more experienced dentists were more likely to oppose implantation performed by undergraduates. The most preferred implantation method was implant insertion using a digitally fabricated drilling template, after surgical flap elevation.

**Conclusions:**

Students and graduates preferred a comprehensive undergraduate education that included implant dentistry. Dentists working in private practice, and especially dentists working as university educators, were critical towards the integration of implant-related learning content into undergraduate education. The intention of medical education is to impart knowledge to students and to prepare them for life-long learning and continual professional development after graduation. Thus, an undergraduate dental curriculum that provides students a solid introduction and knowledge foundation in implant dentistry is recommended.

## Background

The use of dental implants is perhaps one of the most profound changes that have defined modern dentistry, at least for those who can afford it. Although implant dentistry is a relatively young scientific discipline, however, it has expanded exponentially within the span of a few decades and is practiced worldwide [1]. In Germany, the provision of implant-supported restorations among the 65- to 74-year-old age group has multiplied tenfold to 8.1% in the last 15 years [2, 3]. With increased patient awareness and treatment expectations, dental graduates must be cognizant of the indications for implant care and provide predictable treatment [4].

Inevitably and in parallel with the expansion of implant dentistry, the need for standardized and structured implant dentistry education has been recognized through a global consensus among clinicians, researchers, and educators [5–9]. Nevertheless, the definition of an adequate training format has been complicated by the fact that implant dentistry involves competencies from multiple disciplines, including oral surgery, periodontics, restorative dentistry, and prosthodontics.

Expert consensus meetings and opinion leaders throughout the world have repeatedly pointed to the importance of a multidisciplinary, evidence-based education [10–13] prior to practicing implant dentistry. At the 2008 Prague Consensus Conference on Implant Dentistry (Association for Dental Education in Europe), a consensus was reached regarding the procedures within implant dentistry with which the newly graduated dentist must exhibit competence. This reflects the increasing importance of implant dentistry to the dental profession, and that certain aspects of this discipline should indeed be integrated into the undergraduate curriculum [5, 10, 14].

According to the currently available recommendations, undergraduate implant education should include basic aspects of healing and tissue integration, biomechanical and material principles, and prosthetic and surgical skills [11, 15, 16]. As available time within dental curricula is limited, the addition of new learning content necessitates the reduction or complete removal of other previously established content. Therefore, it is still a matter of debate as to whether these recommended implant-related teaching contents should be integrated into the undergraduate curriculum or not [13]. Although dental undergraduates appreciate implant dentistry as something modern and important, specialists in the discipline emphasize the need for advanced surgical skills and clinical experience before the first implants are inserted. Studies have shown that recent graduates, owing to a lack of experience with surgical or implant-related complications, often do not interpret surgical results as more experienced dentists would interpret them [1]. University educators have often emphasized this issue and have thus made efforts to ensure that students in practical treatment courses have acquired the requisite basic clinical skills for each core discipline [13]. This has been expressed by experienced dentists and clinical educators who have stressed that undergraduate time should be reserved for the acquisition of general knowledge and practical skills in the core disciplines and that implant dentistry should be classified as a postgraduate endeavor.

Thus, the aim of the present study was to evaluate the perspectives of undergraduate students, clinical educators, and experienced dentists in terms of the importance of theoretical and practical teaching content in implant dentistry in undergraduate dental education. The specific objective was to determine whether a consensus would exist concerning aspects of theoretical knowledge, implant position planning, implantation, prosthetic treatment procedures, postoperative care, and prerequisite experiences that should be provided in undergraduate dental education.

## Methods

This study used a cross-sectional, online survey-based, exploratory design. Institutional Review Board approval was provided by the Ethics Committee of the Martin Luther University Halle-Wittenberg. The survey instrument was designed based on the recommendations of the 2008 Prague Consensus Conference on Implant Dentistry [5] and the authors’ perspectives.

A 27-item questionnaire was designed. Five questions pertained to demographic data and nine questions pertained to teaching content in implant dentistry. Opinions regarding prerequisites before the first implantation and the prioritization of conventional and modern teaching contents in dental education were also obtained. The survey questions were pilot-tested on site by five experienced dentists. The survey document consisted of a short introduction and informed consent for electronic approval. It was hosted on the “SoSciSurvey” online platform. Before releasing the survey online, an invitation for participation was published in the *Journal of Dental Implantology* [17]. The survey was available online between June 1, 2018, and December 31, 2018, on the internet platform of the *Journal of Dental Implantology*, the official journal of the German Society for Implantology. A survey link was distributed through email, and social media networks were used to invite participants to complete the survey and share the electronic survey link with potentially interested dentists. No incentives were provided for survey completion.

## Results

**Fig. 1:**
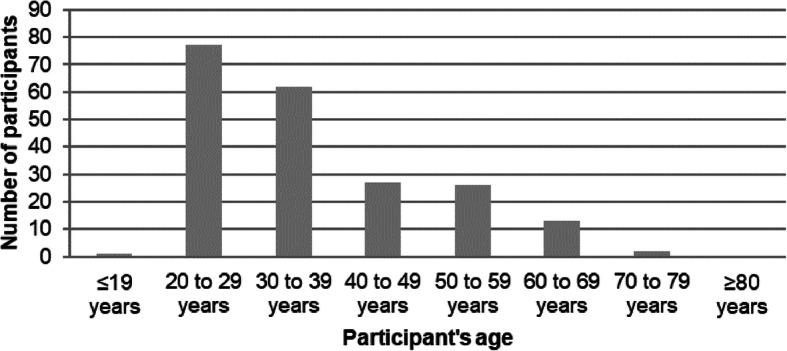
Age distribution of the survey participants

Among a total of 678 German participants who accessed the survey link, 208 (females, 101 [48.6%], males, 107 [51.4%]) completed the survey, yielding a response rate of 30.7%. More than one-half (66.8%) of the participants were aged between 20 years and 39 years (Fig. 1).

**Fig. 2:**
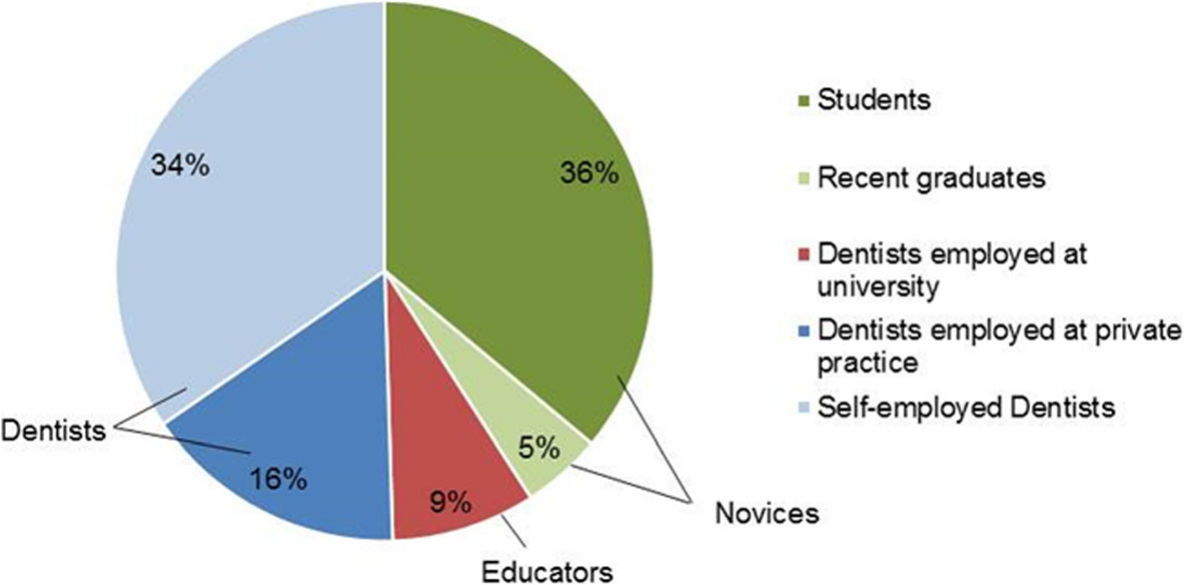
Participants’ current employment

Participants were categorized into three groups based on their current employment: (1) “novices” (undergraduates and dentists who were within 2 years of graduation, *n* = 85 [41%]), (2) “educators” (dentists providing university-based dental education, *n* = 18 [9%]), and (3) “dentists” (employed and self-employed dentists working in a private practice, *n* = 105 [50%]) (Fig. 2). Survey results were statistically compared between the three groups, and consensus was defined as ≥ 60% of each group having selected the same response. At the time of the survey, 107 (51.4%) participants had no prior experience of implant placement, and 42 participants (20.2%) had inserted more than 500 implants over the course of their career.

### Implant dentistry as a part of the dental curriculum

The systematic teaching of essential theoretical and methodological content in implant dentistry was supported by 99.9% of the novices, 88.3% of the educators, and 81.2% of the dentists. Novices considered the introduction of the digital workflow as desirable, whereas dentists preferred a prosthetically oriented educational program that was ideally supervised by a surgically experienced specialist in prosthodontics.

**Fig. 3:**
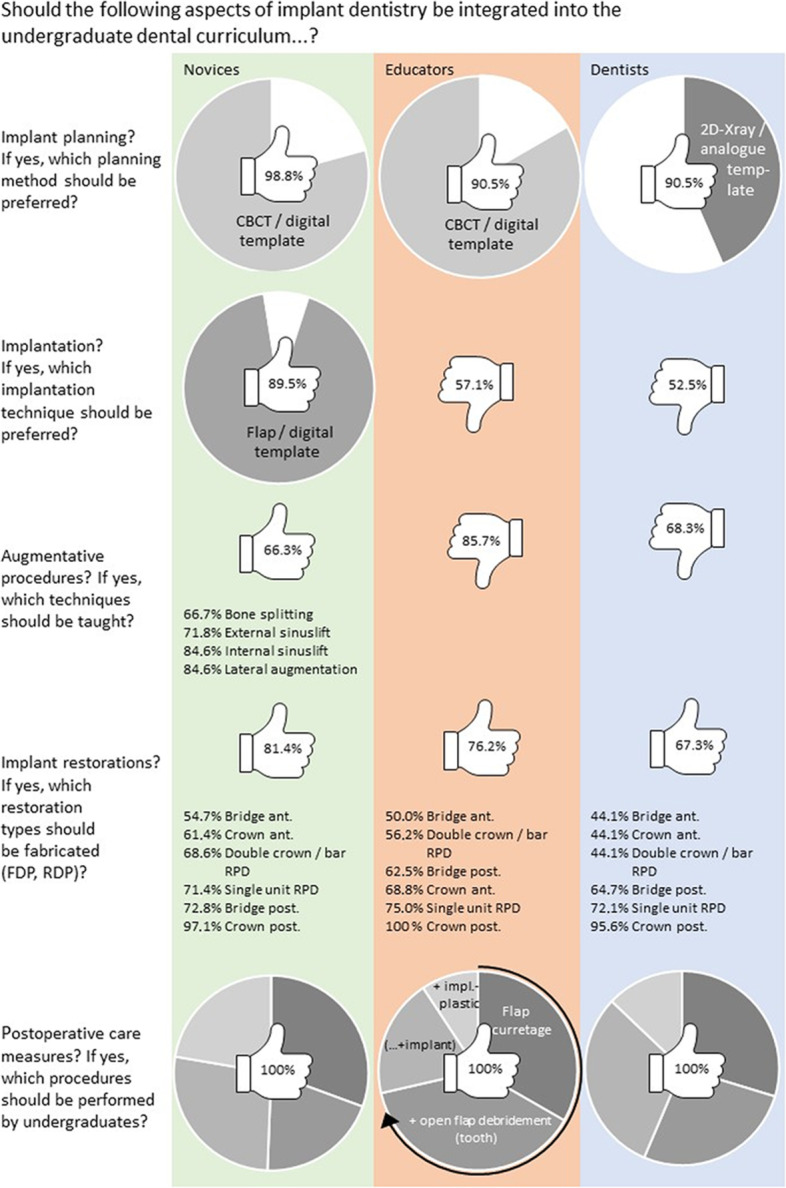
Participants’ answers to questions about content that should be taught in a dental curriculum. The percentages within the thumbs indicate acceptance (thumb up) or rejection (thumb down) within the three groups (i.e., novices, educators, and dentists). CBCT, cone beam computed tomography; FPD, fixed partial denture; RPD, removable partial denture; ant., anterior (i.e., incisors, canines); post., posterior (i.e., molars, bicuspids)

The answers to questions regarding practical teaching content (e.g., implant planning, implantation, augmentation, implant restoration, and aftercare) that may be included into a dental curriculum are listed in Fig. 3. The majority of novices appreciated all practical teaching content, whereas most educators and dentists rejected implantation and the performance of augmentative measures by undergraduates. The more implants the participants had already inserted, the more they disapproved of implant planning and implantation by undergraduates (*p* < 0.001, based on Pearson’s chi-squared test). Regarding augmentative and postoperative care measures, responses were not influenced by group affiliation or previous experience with implants (*p* > 0.05, based on Pearson’s chi-squared test).

Regarding implant restoration, a consensus existed between the groups for crown and bridge fabrication in the posterior region and for overdentures fixed on single unit elements such as ball attachments (Fig. 3).

### Practical surgical prerequisites before performing the first implantation

**Fig. 4:**
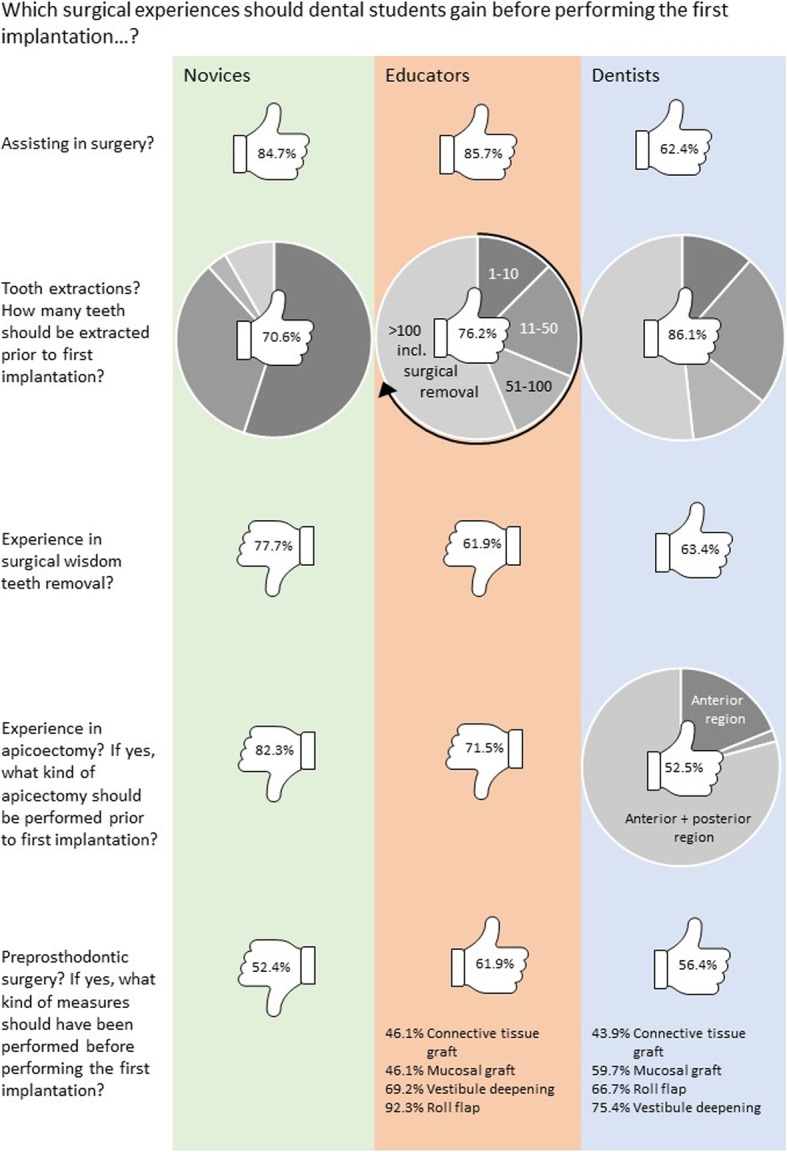
Answers to questions about surgical prerequisite experiences before performing the first implantation. The percentages within the thumbs indicate acceptance (thumb up) or rejection (thumb down) within the three groups (i.e., novices, educators, and dentists)

The answers regarding potential practical surgical prerequisites that should be met before the first implantation are listed in Fig. 4. A consensus existed between the groups regarding assisting in surgery before the first implantation. The importance of surgical skills was evaluated differently, depending on group affiliation and implant experience. The more implants the participants had previously inserted, the more they considered enhanced skills in tooth extraction (including surgical removal of the wisdom teeth) and apicoectomy as necessary before performing the first implantation (*p* < 0.01, based on the Pearson’s chi-squared test). Regarding the necessity of preprosthodontic surgery experience, group affiliation and implant experience did not influence the chosen response (*p* = 0.271, based on Pearson’s chi-squared test).

### Practical prosthetic prerequisites before performing the first implant treatment

**Fig. 5:**
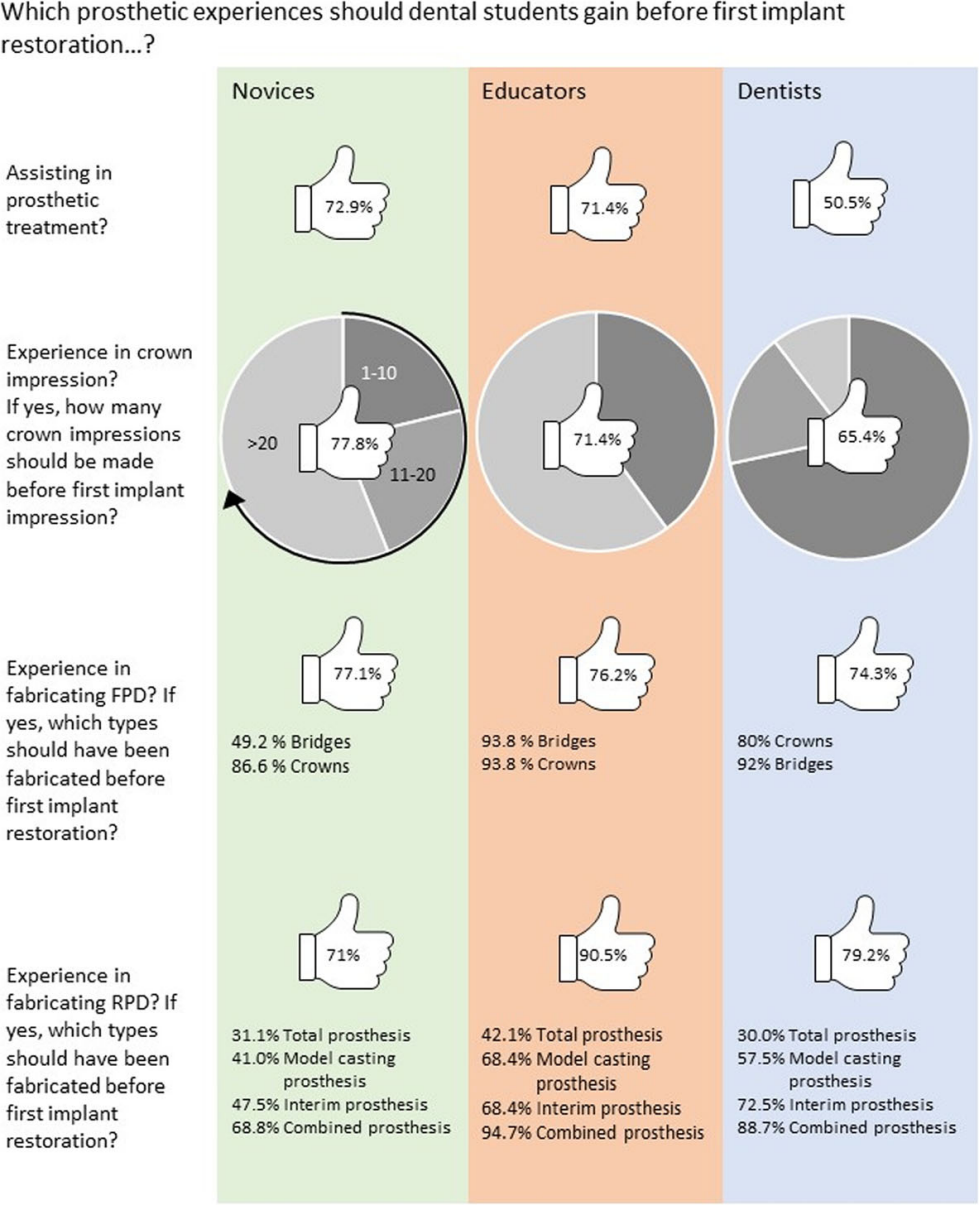
Answers to questions about prosthetic prerequisite experiences before performing the first implant restoration. The percentages within the thumbs indicate acceptance (thumb up) or rejection (thumb down) within the three groups (i.e., novices, educators, and dentists). FPD, fixed partial denture; RPD, removable partial denture

The answers regarding potential practical prosthetic prerequisites that should be met before performing the first implantation are listed in Fig. 5. A consensus existed between the groups regarding assisting in prosthetic treatment before the first implant restoration. Regarding the experience in crown impression as a prerequisite for the first implant impression, a fewer number of impressions was deemed necessary by dentists than by novices and educators (*p* < 0.01, based on Pearson’s chi-quadrat). Most participants appreciated having experience in fabricating removable partial dentures before the first implant treatment. Experience in the fabrication of interim prostheses was considered less important than that in the fabrication of other denture types. Compared to educators and dentists, novices considered experience in the fabrication of removable dentures less important (*p* < 0.01, based on Pearson’s chi-quadrat).

### Prioritization of teaching content

**Fig. 6:**
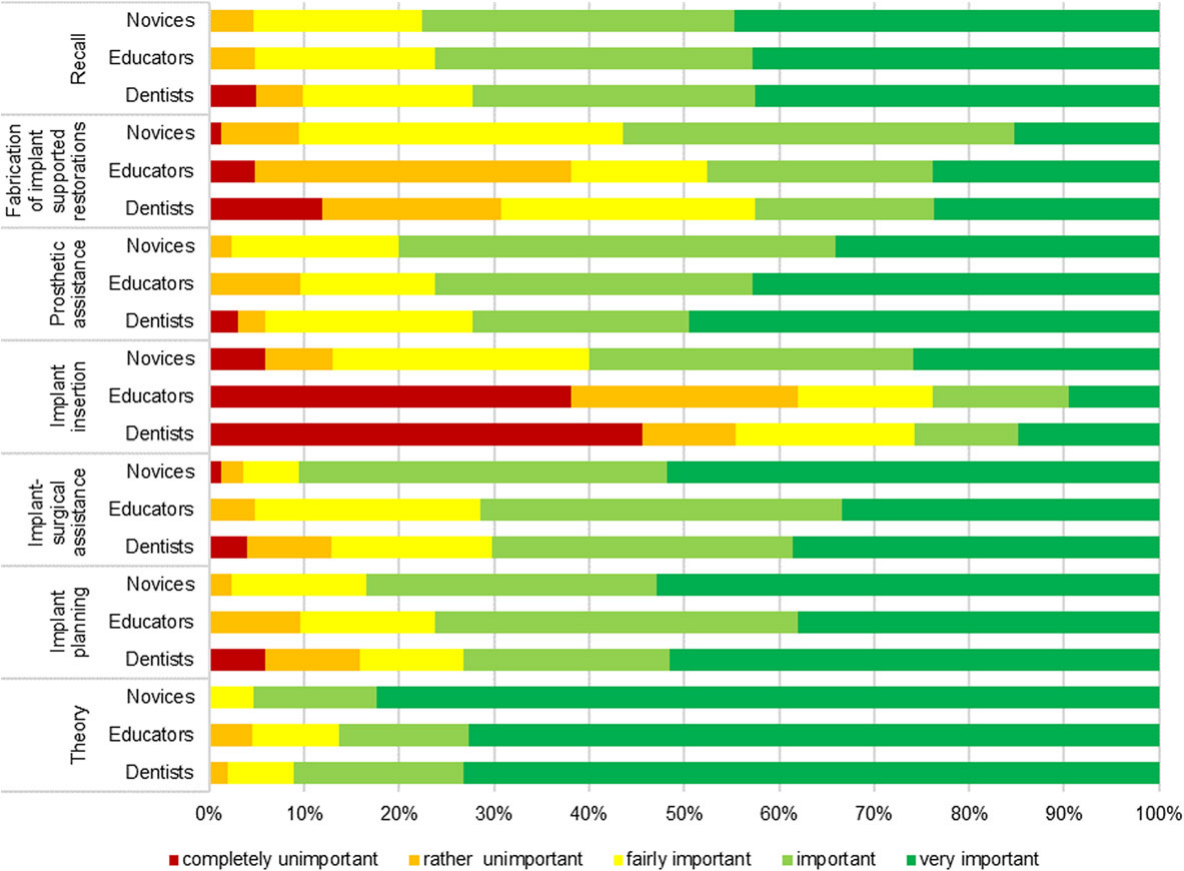
The importance of being taught content about implant dentistry, as ascribed by participants. The participants scored each learning category as “completely unimportant,” “rather unimportant,” “fairly important,” “important,” or “very important”

All three groups considered a comprehensive education in the theoretical aspects of implant dentistry and postoperative care as important; however, the appreciation of the importance of prosthetic and surgical content in the dental curriculum differed considerably (Fig. 6). This was especially so for “implantation by undergraduates,” which was considered as “rather unimportant” and completely unimportant” by dentists and educators.

## Discussion

As a multispecialty domain, implant dentistry involves competency in oral surgery, periodontics, restorative dentistry, and prosthodontics. Thus, classifying implant dentistry as a subspecialty of any existing formal dental specialty is difficult [18, 19]. The resulting multiplicity of professional stakeholders, along with the perceived high commercial value, may have together contributed to a rather unregulated surge in actual implant practice that has far outpaced formal university-based training [20]. Professional societies have attempted to establish postgraduate curricula, but the relative lack of university-based training has often been largely substituted by informal, short-term, unstructured, and industry-initiated educational opportunities [21]. The educational value of these industrial promotional events often remains questionable because implant dentistry is mostly reduced to a mechanistic procedure that can be simply handled if the right system is used. To manage this threat, some expert committees have recommended that basic aspects (i.e., healing and tissue integration, biomechanical and material principles, and prosthetic and surgical skills and procedures) should be a formal part of the undergraduate curriculum [4–6, 12, 15]. Whether this should also include competency in implant surgery remains a matter of debate [7]. The findings of our study confirm that the attitudes concerning the importance of implant dentistry for dental students vary widely and may depend on the level of clinical experience in implant dentistry and the vested interest in the provision of undergraduate dental education.

### Group assignment

Participants were assigned into three groups (i.e., novice, educator, and dentist), based on their level of educational and clinical experience. The rationale for this assignment was to present the different viewpoints of the targeted learners, providers of undergraduate education, and experienced dentists. University educators would logically be expected to provide the best insight into the possibilities and limitations of dental education. University educators are highly cognizant of these issues and thus make efforts to design curricula with the goal of ensuring that students acquire the requisite basic clinical skills for the core dental disciplines. However, this task is complicated by the fact that undergraduates treat different patients with a wide range of oral problems that require treatment with varying levels of difficulty. However, they are expected to have acquired a standardized knowledge base and a set of practical skills before graduation [13]. Thus, even without taking into consideration the discipline of implant dentistry, the organization and supervision of traditional undergraduate practical treatment courses remain a significant challenge, and university educators are often resistant to proposed changes and innovations. To account for this in our survey, the dentist group was split into educators and dentists working in universities, and dentists working in private practices. The dentist group had the highest proportion of participants with an enhanced level of implantation experience (> 500 implantations performed).

### Theoretical knowledge

A consensus existed between the three groups regarding the teaching of theoretical knowledge and skills. This consensus is in accordance with recommendations from the literature for sound knowledge in clinical information gathering, diagnosis and treatment planning, establishing and maintaining oral health, surgical procedures, periodontal management, restorative and prosthodontic management, and health promotion [5, 11, 16]. The requirement for educational program supervision by an experienced specialist in prosthodontics has been emphasized and is current practice in some dental schools in North America [18, 19]. This is in line with the requirements of a synoptic treatment concept, with implants being one component of comprehensive rehabilitation.

### Implant planning by undergraduates

One question was whether implant planning should be a part of undergraduate dental education. In general, this proposition was supported by most participants in each group.

With regard to the preferred implant planning methods, novices and educators favored the use of cone beam computed tomography (CBCT). However, dentists in private practices preferred conventional implant planning by using standard radiographs (i.e., panoramic and periapical radiographs), and the fabrication of an analog drilling template in the undergraduate curriculum. The disagreement reflects the current debate on the differing guidelines concerning the necessity of CBCTs for implant planning [22]. The guidelines’ divergence was interpreted as an influential factor for health professionals to ignore these recommendations. To date, German dentists have no current guidelines. The CBCT recommendation regarding implant planning from 2013 has been under revision since 2018.

In general, the preparation of clinical materials in the laboratory (e.g., the fabrication of drill templates) and clinical implant planning received very positive feedback from the undergraduate students, and these activities had a positive influence on the graduates’ future plans to perform implant therapy [4, 23, 24]. However, Fortes et al. [25] reported that implant planning by novice and experienced dentists differed if standard radiographs were used instead of CBCT images. This finding may be because of the fact that, compared to conventional planning methods, modern planning software anticipates several planning steps in advance and is able to provide a higher level of safeguards for a novice. Thus, based on these considerations and the limited amount of available time in undergraduate dental curricula, implant planning using CBCT may be suitable in undergraduate education.

### Implantation by undergraduates

Implantation by undergraduates was not supported by 57.1% of educators and 52.5% of dentists. By contrast, most (89.5%) undergraduate students supported the teaching of this procedure. The educator group interestingly had the greatest proportion of critics. This finding may be because of the fact that implantation by undergraduates requires direct supervision by an experienced instructor and a wide range of tools, which are often expensive. An experienced clinician is also required for screening and selecting suitable patients. Moreover, educators may be tasked to teach in areas of the curriculum and to supervise clinical procedures in which they have limited experience [13, 24, 26].

The results of previous studies [27] have shown that implantation using CBCT-based digitally fabricated drilling templates achieves good results that are independent of an operator’s experience. Thus, from a scientific point of view, this method may be suitable, even for novices. In addition, patients prefer the comfort of computer-guided flapless surgery and positively evaluate implantation performed by undergraduates [28–30]. However, experienced surgeons often criticize that less experience, in combination with an enhanced sense of safety, leads to a lack of awareness regarding potential intraoperative complications and a false sense of security on the part of the student [1]. This risk may be countered by undergraduate students’ management of more complex and demanding implant treatments in university hospital out-patient departments. The clinical results of different implant educational programs implemented at different universities worldwide are acceptable and have success rates ranging from 92.2 to 98.8%, using different implantation techniques (e.g., drilling and orientation templates) [15, 28, 31–34]. This outcome is comparable with that of other studies in which surgeries were performed by experienced surgeons [35, 36], and suggests that undergraduate students are capable of performing basic implant surgery under supervision, provided that straightforward cases are assigned to them and thorough preclinical training is provided beforehand. Thus, these results provide support for the integration of implantation into the undergraduate curriculum.

### Bone augmentation by undergraduates

Educators, dentists, and one-third of the novices did not support the integration of bone augmentation into the undergraduate curriculum. This assessment of augmentative measures as a domain of postgraduate training and specialist management is in accordance with recommendations reported in the literature [11, 26]. In most studies reporting undergraduate implant curricula, the necessity for bone augmentation has been described as an exclusion criterion [28, 31, 32].

### Implant restorative treatment by undergraduates

A consensus existed regarding the question of whether undergraduates should perform implant restorations. Novices appreciated the opportunity to perform a diverse range of restorations, including those in the esthetically demanding anterior region, whereas experienced dentists disapproved of undergraduates performing complex treatments. De Bruyn et al. [7] evaluated undergraduate dental implant programs in Europe and found that only 44% of programs permitted undergraduate students to perform prosthetic restorations by using implants. These procedures were primarily limited to single crowns on molars (28%) or bicuspids (33%), and only 16% of cases involved implants in the esthetic zone [7]. Between 9% and 23% of cases involved mandibular overdentures retained by two implants. These findings are consistent with the results of American and Canadian surveys [13, 18, 19]. Most experts agreed that only straightforward restorative or prosthetic procedures should be a part of an undergraduate curriculum [11, 13], thus correlating with the attitudes of the dentists participating in our study. The fact must also be acknowledged that the enthusiasm demonstrated by undergraduate students, in combination with their lack of experience, may often result in their underestimation of the difficulty and complexity of cases requiring implant therapy and the need for specialist referral.

### Postoperative care provided by undergraduates

With regard to postoperative care, no relevant differences existed between the attitudes of novices, educators, and dentists. This finding is in agreement with previous recommendations, which have stressed the importance of knowledge and skills in aftercare measures in the undergraduate curriculum [11, 13]. With regard to the aging demographic profile in Germany and worldwide, and the recent surge in implant treatments, it seems reasonable to train undergraduates in postoperative care and long-term maintenance, as they will be confronted with this task immediately after graduation [2, 3].

### Practical surgical prerequisites before the first implantation

In contrast to the novices, the dentists and educators emphasized the need for advanced surgical skills and clinical experience (i.e., increased number of tooth extractions, operative removal of third molars, apicoectomy, preprosthetic soft tissue surgery) before performing the first implant insertion. This finding supports that of a previous international study that formulated the assumption that, on account of a lack of experience regarding surgical or implant complications and demands, graduates do not interpret surgical results in the same manner as more experienced dentists [1]. Deficiencies in the ability to discriminate normal healing and anatomical structures from pathology may lead to a novice’s failure to detect less-than-ideal treatment outcomes.

Of note, educators did not rate surgical skills as highly as dentists did. One explanation may be that enhanced surgical skills training in undergraduate education is simply impractical for the predetermined time provided. However, the demand for enhanced practical skills were correlated with the implantation experience of the participants. Dentists working in private practices constituted one-half of the survey participants and more than one-fifth of the participants were highly experienced in implant therapy (i.e., they had inserted more than 500 implants over the course of their career). This factor may have influenced the results.

### Practical prosthetic prerequisites before first implantation

Compared to dentists, the novices and educators interestingly evaluated assisting during implant prosthetic treatment and experience with crown impressions before performing the first implant treatment as more important. This finding may be because conventional prosthodontics has had an increasingly important role in recent decades in the German undergraduate dental curriculum and because crown impression-taking has routinely been emphasized from the first practical phantom courses up to the final examinations [37]. As the performance of crown impressions differs from that of implant impressions, the former procedure may not necessarily be seen as a prerequisite for the latter procedure. In a crown impression, the gingival margins have to be compressed to prevent gingival fluids/blood from spilling out of the gingival sulcus over the preparation margin; an implant impression requires the correct placement of the correct impression post. By contrast, an agreement was found between educators and dentists regarding the necessity for undergraduates to have obtained experiences in the fabrication of tooth-supported fixed and removable dentures before they started to fabricate implant restorations.

Several recommendations exist in the literature regarding surgical and prosthetic simulation procedures that should be performed on phantom heads or artificial jaws. However, to the best of our knowledge, no recommendations exist pertaining to the types of conventional dentures that undergraduate students should provide to patients as a prerequisite, before starting their first implant cases. On account that educators and dentists considered this aspect as important, previous experience with specific conventional denture treatment during the planning of future dental (implant) curricula seems reasonable to consider. In the early years, when implant dentistry was mainly applied on edentulous patients, this was accepted as a prerequisite.

### Prioritization of teaching content

Both educators and dentists considered implantation performed by undergraduates much less important than teaching basic theoretical knowledge, postoperative care and maintenance, and the prosthetic restoration of implants. This finding is supported by the recommendations of previous studies that have advocated the need for solid theoretical foundations and the restoration of simple “straightforward” cases, based on the Simple, Advanced, Complex (SAC) classification, which has been ascribed a higher priority than the implantation itself [6, 13].

However, it has to be emphasized that when the daily practices of graduates from a dental school with and without extensive implant education (laboratory and clinical experience in implant placement and restoration) are compared, graduates who had clinical implant experience during their undergraduate education were found to have performed twice as many implant restorations in their practice; placed more dental implants; referred more patients for specialized surgery; and participated more often in continuing education in implant dentistry [38]. The objective of health sciences education is to deliver rote knowledge to students and to prepare them to overcome new challenges and clinical problems over the course of their careers as dentists. Therefore, we recommend that a solid foundation in implant dentistry be fully integrated as a minimal standard within an undergraduate dental curriculum in countries with the appropriate resources [39].

### Limitations of the study

The current study has several limitations. It was based on an anonymized online survey. Thus, the participants’ answers could not be independently verified. In the study cohort, the number of educators was smaller than that of dentists and novices. In addition, the survey questions pertaining to teaching content only took into consideration the overarching issues, based on the recommendations of the 2008 Prague Consensus Conference on Implant Dentistry [5]. Future studies are necessary to define precise learning objectives. Moreover, undergraduates generally experience stress related to difficulties in meeting procedural clinical requirements; therefore, additional studies are required to evaluate which teaching and learning components within existing curricula can be reduced or replaced with implant dentistry teaching content [40]. Finally, the transferability of our study’s results may depend on the financial resources countries have in order to implement implant curricula into the dental curriculum. Especially in developing countries, many impediments such as the lack of financial resources, lack of qualified faculty, or inadequate curriculum time make implementing implant dentistry in undergraduate curriculum difficult [41].

## Conclusions

The students and recent graduates who participated in this study preferred a comprehensive undergraduate education that would include implant dentistry. By contrast, dentists working in private practice and dentists working as university educators, in particular, were critical towards the inclusion of this discipline in the curriculum. A positive consensus existed between the three participant groups with regard to theoretical education, assistance in surgery and prosthetic procedures, implant planning and restoration in straightforward cases (i.e., posterior single crowns and bridges, overdentures on nonsplinted implants), and postoperative care and maintenance. A negative consensus existed for integrating bone augmentation into the undergraduate curriculum. While implantation was supported by students and recent graduates, the disapproval expressed by practicing dentists was associated with increased experienced in implant dentistry. The most preferred implantation method was implant insertion after flap elevation with the use of a digitally fabricated drilling template. The results of this study suggested that undergraduate dental curricula should at least provide students a solid introduction and knowledge foundation in implant dentistry.

## Data Availability

All data generated or analyzed during this study are included in this published article.

## Abbreviations

CBCT: Cone beam computed tomography
SAC: Simple, Advanced, Complex
